# Alzheimer's disease and its severity reduce MEG responses to unexpected auditory stimuli

**DOI:** 10.1002/alz70856_102668

**Published:** 2025-12-26

**Authors:** Alexandra Krugliak, Mats WJ van Es, Marlou N Perquin, Chun Shen, Haddy Fye, Cara Alcock, Juliette H Lanskey, Melek Karadag, Ece Kocagoncu, Rebecca Williams, Andrew J Quinn, Jemma Pitt, Tony Thayanandan, Stephen L Lowe, Vanessa Raymont, Krish D Singh, Mark W Woolrich, Anna C Nobre, James B Rowe

**Affiliations:** ^1^ University of Cambridge, Cambridge, Cambridgeshire, United Kingdom; ^2^ University of Oxford, Oxford, Oxfordshire, United Kingdom; ^3^ Fudan University, Shanghai, Yangpu District, China; ^4^ University of Birmingham, Birmingham, West Midlands, United Kingdom; ^5^ University of Oxford, Oxford, United Kingdom; ^6^ Eli Lilly and Company, Singapore, Singapore; ^7^ Cardiff University, Cardiff, Glamorgan, United Kingdom; ^8^ Yale University, New Haven, CT, USA; ^9^ University of Cambridge, Cambridge, ‐, United Kingdom

## Abstract

**Background:**

Alzheimer's disease (AD) impairs cognition with synaptic and neuronal loss related to amyloid and tau pathology. Drug development needs sensitive and reliable biomarkers for early phase clinical trials. The effects of AD on brain function can be measured by magneto/electroencephalography (M/EEG). The New Therapeutics in Alzheimer's Disease study (NTAD) aims to test the sensitivity and reliability of M/EEG‐based biomarkers to AD.

The entorhinal cortex and hippocampi are affected early by AD, underlying deficits in associative learning. However, it is difficult to directly measure physiological responses from medial temporal lobe by MEG due to its depth and orientation. In contrast, lateral auditory cortical responses to unexpected stimuli (e.g. mismatch negativity response) are readily detected by MEG. We therefore developed a hybrid task, based on associative learning, with auditory novelty and associative deviants to rapidly presented standard trials: the audio‐visual oddball task (VAB).

**Method:**

We used concurrent M/EEG to measure neural responses to unexpected novel sounds and mismatches of associated image‐sound pairings. Cognitively healthy older controls (*N* = 31) and people with amyloid‐positive mild cognitive impairment (MCI) or early AD completed baseline assessment (*N* = 55). A subset of patients (*N* = 17) were re‐tested after two‐weeks to assess the reliability of the VAB.

**Result:**

A sensor‐level analysis on gradiometers revealed that controls showed a stronger neural response to novel compared to mismatching sounds. In AD/MCI patients the response to mismatching sounds was increased compared to controls, indicating reduced differentiation between novel and learned sounds consistent with deficits in associative learning. This increase in neural response to mismatched sounds in patients related to cognitive deficits (Addenbrooke's Cognitive Examination, ACE‐R). Comparing the VAB measures of the baseline assessment and two‐week scans shows moderate to good reliability. An analysis in source space is in progress.

**Conclusion:**

Our results demonstrate that the MEG responses in the VAB task are sensitive to presence of AD/MCI. With sufficient reliability we propose that MEG is suitable to support experimental medicine studies of AD/MCI.

